# A shared decision-making model in pediatric palliative care: a qualitative study of healthcare providers

**DOI:** 10.1186/s12904-023-01307-0

**Published:** 2023-11-28

**Authors:** Siyu Cai, Lei Cheng, Ruixin Wang, Xuan Zhou, Xiaoxia Peng

**Affiliations:** 1 Center for Clinical Epidemiology and Evidence-based Medicine, Beijing Children’s Hospital, Capital Medical University, National Center for Children’s Health, 56 South Lishi Road, Beijing, 100045 China; 2 School of Nursing, Fudan University, Shanghai, 200032 China; 3 Beijing Key Laboratory of Pediatric Hematology Oncology; National Key Discipline of Pediatrics (Capital Medical University); Key Laboratory of Major Diseases in Children, Ministry of Education; Hematology Center, Beijing Children’s Hospital, Capital Medical University, National Center for Children’s Health, 56 South Lishi Road, Beijing, 100045 China

**Keywords:** Shared decision-making, Pediatric, Palliative care

## Abstract

**Background:**

Pediatric shared decision-making (SDM) is a fundamental part of family-centered care. Pediatric palliative care (PPC) is one of the more difficult fields for healthcare providers when choosing to utilize SDM. However, to our knowledge, there are still few structured approaches of SDM in PPC. We aimed to build a model of SDM in PPC that achieves better care and outcomes for children and their family members.

**Methods:**

This study is a descriptive phenomenology study. Participants included physicians, nurses, and social workers in the PPC team. Participants were individually interviewed face-to-face or via an online meeting software. Data were collected in semi-structured interviews and analyzed using a thematic framework analysis.

**Results:**

In total, 27 healthcare providers were interviewed. The model of SDM in PPC identified three themes, including the participants, the principle and the process of SDM. Decision participants involved the children, parents, the PPC team and others. The decision principle had three sub-themes including type, standard and precondition. The decision process describes the fundamental process of SDM and provides suggestions for mobilizing patients and parents to engage in decision-making and seeking conflict resolution.

**Conclusions:**

This is the first study to develop a SDM model in PPC. This model can provide guidance to PPC teams on SDM practices. In addition, the model contributes to the existing body of knowledge by providing a conceptual model for SDM in the context of PPC.

**Supplementary Information:**

The online version contains supplementary material available at 10.1186/s12904-023-01307-0.

## Background

Pediatric shared decision-making (SDM), a fundamental part of family-centered care, is defined as the process by which healthcare decisions are made through respectful collaborations between clinicians and patients and their parents [1]. SDM can help to serve the best interests of the child and the family, and minimize decision conflicts, as well as to improve compliance, satisfaction and outcomes [2, 3].

SDM is particularly important in pediatric palliative care (PPC) because many decisions depend heavily on the values and preferences of children and families [4]. However, PPC is one of the more difficult fields for healthcare providers when utilizing SDM [5]. Decision-making in PPC is perceived as a long, emotional and challenging journey [6]. The challenges commonly include: (1) Multiple parties: Multidisciplinary teams, the child and the family are involved in making decisions, which can lead to a complex decision process and difficult cooperation; (2) Abilities of children: Children have varied levels of willingness to engage in decision-making due to their interest in the situation and their ability to understand it; (3) Complex medical decision-making: Healthcare providers need to be able to explain the potential options and possible outcomes of complex medical decision-making in lay terms to enable parents to make an informed decision; (4) Emotional burden: Many end-of-life decisions will cause a huge emotional burden on the patient and family, which can make it difficult for healthcare providers to initiate these decision-making; (5) Limited time and resources; and (6) Ethical dilemmas [5, 7–9].

However, to our knowledge, there are still few structured approaches to SDM in PPC. Existing approaches tend to be limited to life support treatment decisions (e.g. artificial nutrition) and are targeted at special groups of patients (e.g. adolescents). A common understanding of SDM in PPC is still lacking [10]. In our study, we strove to build a model of SDM in PPC that achieves better care and outcomes for families and children with life-limiting conditions.

## Methods

### Design

This study is a descriptive phenomenology study. This study aims to construct a general decision-making model, rather than targeting a specific medical decision. Therefore, in addition to the decision-making process, we focus on the principles of SDM practices and strategies to address difficulties. The research method and its reporting follow the Consolidated Criteria for Reporting Qualitative Research (COREQ) [11].

### Sample criteria

Physicians, nurses, and social workers in the PPC team, with at least 3 years of working experience, as well as an abundance of experience in SDM were eligible to participate in the study. A total of 28 healthcare providers were invited, 27 of whom agreed to participate. One physician declined to participate due to limited time availability. Participants were identified through the Pediatric Palliative Care Subspecialty Group of the Pediatrics Society of the Chinese Medical Association. A purposive sampling strategy was applied to ensure a diverse range of participant characteristics (e.g., professional group, gender, and age). Participants could nominate other colleagues to participate. A letter with the objective, methods, and data protection measures of the study was offered to potential participants.

### Data collection

Participants were individually interviewed face-to-face or via an online meeting software (Tencent meeting and Zoom), which took place between November 2022 to January 2023 by SY Cai (MD, female) and L Cheng (PhD, female). Semi-structured interviews, lasting from 36 to 106 min, were audiotaped and transcribed verbatim. Interviews were conducted in Chinese. During the interview, nonverbal information such as expressions and tone were also recorded. Participants were numbered according to their identity and interview order (e.g.: Physician 01). The general information of participants was managed and encrypted by the corresponding author. During the interview, no third person was allowed to be present. No repeat interviews were carried out. Field notes were made after the interview.

The interview guide was developed based on the IP-SDM model and pilot tested in 3 interviews [12]. The interview explored four questions: (1) Please share your experience of SDM; (2) What are the steps involved in SDM in PPC?; (3) What difficulties have you encountered in the process of SDM? And how did you address these difficulties?; (4) Who may be involved in the process of SDM? Participants were asked to add any extra information they considered important at the end of the interview. A detailed interview guide can be found in the supplementary file 1. The sample size was determined by data saturation when no new information was discovered in the data analysis. Data saturation was reached after 23 interviews.

### Data analysis

Thematic framework analysis was performed using Microsoft Word and Excel [13]. Data were coded and analyzed as follows: (1) The researcher became familiar with the data. (2) Two researchers (SY Cai and L Cheng) coded 5 transcripts and constructed an initial coding framework based on the IP-SDM model. (3) Further transcripts were coded to revise and refine the framework until no new themes were generated and the final thematic framework agreed upon. (4) A theme table was created in Microsoft Excel to present the theme, subthemes and supporting quotes. The original words of the interviewees were quoted as much as possible for keywords or expressions and a descriptive analysis was conducted. (5) The themes and codes were discussed, validated and revised in regular meetings to reach a consensus on the themes. The regular meetings were organized by XX Peng and X Zhou, with the participation of core researchers. Descriptions were returned to the participants for review and comment.

### Ethical considerations

This study was approved by the Ethics Committee of Beijing Children’s Hospital Affiliated to Capital Medical University. All of the participants signed the written informed consent.

## Results

**Fig. 1:**
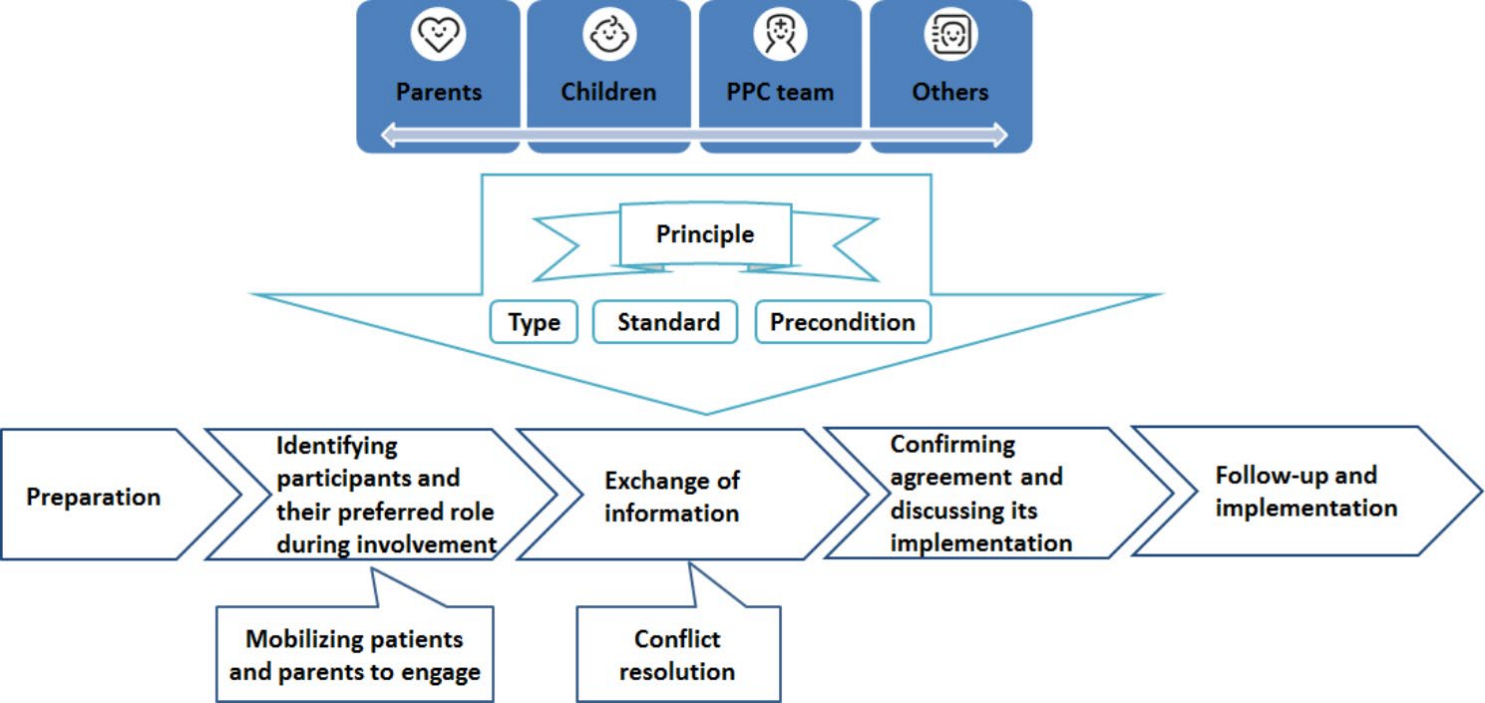
Model of SDM in PPC

In total, 27 healthcare providers were interviewed. Demographic characteristics are shown in Table 1. Model of SDM in PPC is shown in Fig. 1. The model of SDM in PPC identified three themes, including participants, principle, and process of SDM.

**Table 1:** Characteristics of the healthcare providers (N = 27)

Variables		
Age		46 (38, 51)
Gender	Male	4 (14.82)
	Female	23 (85.18)
Occupation	Physician	14 (51.85)
	Nurse	7 (25.93)
	Social worker	6 (22.22)
Academic degree	Doctor	5 (18.52)
	Master	12 (44.44)
	Bachelor	10 (37.04)
Title	Professor	6 (22.22)
	Assistant professor	7 (25.93)
	Attending	11 (40.74)
	Resident	3 (11.11)

Age was described as median (lower quartile, upper quartile)

### Decision participants

The decision participants mentioned by healthcare providers included the PPC team, parents, patients, and others. The core participants in SDM were the multidisciplinary PPC team and parents. Participants stated that children with life-limiting conditions should be involved in SDM to the extent of their developmental level, physical condition, and willingness of themselves and their parents. In addition, participants found that there were other stakeholders who might be involved in SDM, including siblings, grandparents, friends, teachers, spiritual advisors, etc.

Social worker 01: In addition to parents, other relatives may also participate in decision-making, such as grandparents and brothers and sisters.

### Decision principle

The decision principle included three sub themes: type, standard and precondition.

#### Type

Almost all participants (92.59%) considered that all decision contents of PPC were suitable for SDM, including symptom management, psychological, social and spiritual supports, and after-death planning.

Physician 01: I think children and parents can be involved in all decisions… I tried to get them to attend.

Participants generally believed that different decisions require different types of SDM. Participants stated that there were two SDM types in PPC: the recommendation type and the ‘free market’ type. When making treatment decisions, healthcare providers usually adopted the recommendation type. They directly presented the option they believed that was in the child’s best interests and obtained the family’s consent. Sometimes, they communicated in more detail with the family by presenting the pros and cons of each option with a strong emphasis on the preferred option. When making non-treatment related decisions, healthcare providers usually adopted the ‘free market’ type. Healthcare providers would explain to families all possible options, and guide them on how to weigh the relevant advantages and disadvantages.

Physician 01: Treatment decisions are different from other decisions. We need to provide clear suggestions. Otherwise, parents will bear a huge burden when making decisions… For non-treatment related decisions, parents sometimes get confused and don’t know how to choose. We will teach them how to evaluate their options. We will not make decisions for them, but guide them on how to make decisions.

#### Standard

Participants stated that the SDM of PPC relied on the ‘best interests’ standard. In addition, participants believed that, while the child’s best interests were intended to be the overriding factor, healthcare providers should also consider the interests of the family as a whole. Participants mentioned that sometimes they strived to blend priorities and sought some degree of equipoise of the interests of both children and their families.

Physician 02: I think care should be patient-centered. Act in the best interests of the child…We also need to protect the interests of parents. Parents need to move on with their lives after the patient has passed away. We need to ensure that the decisions we make do not harm them.

#### Precondition

Participants emphasized that the precondition for stakeholders to participate in SDM was that they fully understood the children’s condition and reached a basic consensus with the PPC team on the overall care goal.

Physician 02: Parents need to know the current condition of the child. We also need to counsel parents on what PPC is and what our goal is. We should make sure that we are all on the same page.

### Decision process

The decision process describes the fundamental process of SDM, as well as identifies coping strategies for certain difficulties. The fundamental process included five steps. The difficulty of the second step was how to encourage parents and children to participate in decision-making. The difficulty of the third step was how to resolve conflicts. For these difficulties, participants provided their own coping strategies.

#### Fundamental process

The fundamental process of decision-making is an iterative process that needs to be completed for each decision. Participants emphasized that, in clinical practice, SDM might not always fall neatly into the sequential steps as follows.

Physician 06: The process is not always standardized and we need to make adjustments according to the actual situation. Sometimes the exchange of information may take many rounds, or there may be decision-making participants leaving or joining midway.

- Preparation.

Participants mentioned that, before communicating with the family, the PPC team should hold a multi-disciplinary team meeting to: (1) assess the experiences and needs of the family; (2) determine the decisions to be made and their feasible options; and (3) develop communication strategies. Participants suggested that the team needed to reach a unified understanding of the goals of care and the recommended option, and ensure the consistency of the information provided by the different healthcare providers. When the decision required the collaboration of a multi-disciplinary team, team members needed to discuss each member’s unique role, skills and capabilities, and to discuss how to share responsibilities.

Physician 08: Before communicating with parents, we need to reach a consensus. We will hold an internal meeting to determine the content and strategy of communication, as well as the division of labor.

b. Identifying participants and their preferred role during involvement.

Participants found that the form and extent of the child and parents’ involvement could vary widely; from no involvement to dominance, and from a direct to indirect influence. Therefore, participants suggested understanding the willingness and preferred role of the child and parents to participate in SDM and developing individualized ways for them. Children’s participation required the consent of both parents and the children themselves. In addition, the PPC team could explore whether the family wanted other relatives or friends of the child to participate in the decision-making process. Before formal communication, healthcare providers should assess the deciding participants’ understanding of their children’s condition. Participants mentioned that the factors influencing the individuals’ roles in the SDM process included, being the dominant voice of the family, the family culture, the degree of emotional burden on the individual, and their respective level of knowledge pertaining to the illness.

Nurse 07: There are significant differences in the ways parents and children participate in decision-making… The parents’ educational level and family atmosphere all have an impact.

Physician 03: Regarding whether the child participates in decision-making, I will ask the parents first. If the parents agree, I will ask the child. I will also ask the family if they want other people to participate in (SDM).

c. Exchange of information.

Participants stated that communications involved participants’ values, tailor-made information on the pros/cons of each option, and how these decisions fit with the child’s and family’s preferences, values and resources. PPC teams could start with topics about values and goals, which are the basis for decision-making. Participants suggested that the PPC teams explain not only what they believe is the best course of action, but whether this recommendation is supported by evidence and if not, on what basis this recommendation is being made. When evidence was lacking or existing evidence was contested, healthcare providers should acknowledge the uncertainties.

Participants emphasized that healthcare providers needed to provide information, especially medical related professional knowledge, in a language that children and parents could understand. Furthermore, healthcare providers needed to constantly check other participants’ understanding of the information provided.

Physician 04: Regarding palliative sedation, I will tell them the advantages and disadvantages, such as less time to communicate with children. At the same time, I will explore the values and preferences of parents and children and think about how we can meet their preferences or wishes…We need to talk to them in terms they understand.

Physician 01: There’s very little research and a lot of uncertainty, and we need to acknowledge that.

Participants noted that it was difficult for parents to make rational decisions when they showed strong emotional responses. In addition, parents needed enough time to absorb information, ask questions, reflect on the options and build consensus within the family.

Social worker 01: When they are in an emotional state, or they need time to process information, we will give them enough time.

Participants mentioned that they regarded advance care planning documents as important tools to guide their communication. In addition, families used advance care planning documents to understand what decisions might need to be completed in the future, and to think, communicate and record decisions. Participants emphasized that the process of communication and decision-making was more important than signing documents.

Physician 12: I use it (advance care planning documents) as a blueprint for discussion. It can guide my communication.

d. Confirming agreement and discussing its implementation.

After full negotiation, an agreement can be confirmed. Participants mentioned that the PPC team might hold another meeting to discuss the implementation plan of the decision and convey important information to the members of the teams who did not participate in the decision.

Social worker 01: We will hold another meeting to discuss the implementation details, and all relevant staff will participate.

e. Follow-up and implementation.

Participants noted that end-of-life decisions were sometimes made in advance, and families might change their decisions, therefore regular follow-up needed to be arranged. Finally, the decision was implemented according to the plan.

Nurse 05: The condition of children is changing, and families’ decisions will also change accordingly.

#### Mobilizing patients and parents to engage

- Providing information support and decision coaching.

The participants emphasized the importance of providing information support, including the benefits and processes of SDM, as well as children’s rights, abilities and methods to participate in decision-making. In addition, participants emphasized that many families needed guidance on how to inform the children of their illnesses and involve them in SDM. Healthcare providers also needed to provide decision coaching to guide parents to reflect on decisions and build skills in deliberation and communication.

Social worker 02: We should educate parents about the benefits of SDM. In addition, we should help them realize that children have the need to be informed and participate in decision-making.

b. Creating partnerships.

Participants found that families who created partnerships with the PPC teams were more willing to participate in SDM. It was more difficult to establish partnerships with children than adults, which required more time and cooperation of multidisciplinary teams. Children’s most trusted team members were usually those who spent a long time with them, meaning they could become a bridge for other team members to build strong bonds with children.

Nurse 03: We need to build a trusting relationship with the family. We can quickly establish contact with adults. But it takes a long time for children to be willing to talk to you.

c. Respecting children’s decision-making ability and willingness.

All participants encouraged children to participate in SDM as much as possible. Participants noted that healthcare providers should involve children in a personalized manner. Some participants believed that although age was an important factor affecting decision-making ability, family and social culture, as well as personal experiences also strongly influenced children’s decision-making ability and willingness. Participants found that children’s willingness to participate in decision-making was very different. Some children wanted to actively participate in SDM, even leading the decision-making process; some children wanted their parents to make decisions for them; some children preferred to not be actively involved in the decision-making process, but rather they desired to receive information and voiced their preferences. Participants suggested that when children express questions or ideas regarding treatment, care and future planning, healthcare providers could use this opportunity to evaluate the ability and willingness of the children and use it as a trigger to invite the child to participate in decision-making.

Social worker 01: We find that age may not be the most important factor; in some cases a 5-year-old child might have the ability to participate in funeral planning, whereas a 10-year-old child might not.

d. Beginning with simple decisions.

Participants suggested beginning with simple decisions (such as daily life planning and comfort care), and gradually transiting to complex decisions (such as medical decisions). Making a simple decision could make families realize the benefits, feasibility and importance of SDM.

Physician 12: We will begin with simple questions, something easy to think about and answer.

e. Making the decision with an appropriate timing.

Some participants found that plans made prematurely might not be applicable at the time of use because children’s conditions have changed. Therefore, participants argued that it was not always better to make decisions earlier in the disease trajectory, and they believed that different decisions had different appropriate timing. Some participants suggested judging the timing of each decision based on triggers. Triggering factors include changes in the condition of the children, related questions raised by the children and their families, etc.

Social worker 01: Each decision has its own appropriate time. We need to seize the opportunity. For example, parents were not willing to make decisions about funeral affairs too early.

#### Conflict resolution

Participants stated that deciding participants might disagree on the best course of action. They might differently view the problems, benefits and risks. Participants offered some strategies on how to move the conflict dynamic towards a positive outcome.

- Identifying the basic reason of the conflict.

Participants believed that understanding the basic reason behind conflicts was an important way to resolve them. Conflicts could result from a difference in values, goals, priorities, perceptions, and identities. Furthermore, due to the negative emotion and lack of acceptance of a poor prognosis, parents might become narrow-minded about what is best for their child, such as having biases toward initiating more interventions rather than focusing on comfort. Participants suggested that viewing issues from the parents’ perspective would help them understand their choices.

Physician 01: I will ask parents: “You are particularly reluctant to use morphine. What are you worried about?“ Once we can understand the source of a problem, we will better know how to solve it. We need to explore its underlying causes.

b. Seeking common goals among all parties.

Participants stated that siding with one party would lead to further tension. Participants suggested seeking common goals and concentrating on areas of shared interest, which provided a solid basis for conflict resolution.

Nurse 06: We ask parents to focus on their child, to think about what our common goals are and how to do the best for the child.

c. Understanding that the resolution process is more important than the result.

Participants noted that healthcare providers should understand that not all conflicts could be resolved, especially if the parties have longstanding conflicts. Nevertheless, important information obtained during communication, such as values and preferences, should still be recorded. Participants stated that SDM means a shared process, not necessarily a shared decision. In the process of conflict resolution, more attention should be paid to the process of communication and mediation rather than the results.

Physician 06: Sometimes we can’t eliminate the differences completely. Our goal is to improve their understanding of decision-making and mutual recognition in the process of communication.

## Discussion

Families and children with life-limiting conditions need to make a wider range of decisions, including treatment decisions, death location, social supports, and so on, which presents emotional response, stress and moral dilemmas [14, 15]. SDM is promoted as the gold-standard in PPC [16]. This study developed a model of SDM in PPC based on the experiences and perceptions of PPC healthcare providers.

This study was designed to build a model that could guide clinical practice, therefore we focused more on strategies to address obstruction rather than a comprehensive understanding of the various levels of barriers. Previous research has shown that the most frequent barriers in SDM of the pediatric field were features of the options, poor quality information, parent/child emotional state, power relations, and insufficient time [9, 17]. These barriers also exist in SDM practice in the field of PPC. Further research is needed to comprehensively explore the barriers and facilitators of SDM in PPC, in order to further promote the clinical practice.

Beyond the barriers to universality, the biggest challenge for SDM in the PPC field is how children participate in decision-making. SDM in pediatrics is triadic because children may be as much, or even more involved, as the parents. Although children’s participation in decision-making is considered an essential component of quality of care, healthcare providers and parents usually do not involve them in the decision-making process [10]. The reasons include underestimation of a child’s capabilities, presumed complexity and uncertainty of the issue, and protectiveness of the child from reality [18]. This study provides some suggestions for promoting children’s participation in decision-making, and the effectiveness of these suggestions needs further verification.

A child’s capacity to make decisions is not well-defined in existing literature; furthermore, participants used in research tend to be older aged children [19–21]. Participants in this study encouraged children to participate in decision-making as much as possible and believed that children’s participation should be determined according to their actual abilities and willingness, rather than their age. This opinion challenged the belief that age is the main barrier to involvement in decisions, and lends some credence to the assertion that children can also be active decision makers in PPC. As Alderson et al. found, rather than age, other factors such as experience, relationships and values affect children’s involvement in decision making [22]. Alderson and Montgomery also put forward that all children could be included in decisions and that their participation is a stepped process [23].

Given the broad topic of pediatric end-of-life issues and the multiple stakeholders involved, the inter-professional approach was deemed the key point of SDM in PPC [24, 25]. PPC involves professionals from various disciplines, such as physicians, nurses, psychologists, social workers, and chaplains. Each discipline contributes unique expertise and perspectives, allowing for a more holistic, comprehensive and individualized care plan [26]. Le´gare´ et al. developed and validated a conceptual model for an inter-professional approach to SDM in primary care [12, 25, 27]. This study is guided by the model above and provides a theoretical basis for multidisciplinary SDM practices in PPC. Although this study emphasized the importance of information exchange within the team and multi-disciplinary team meetings, we did not explore the experiences of non-core members of decision-making (such as psychologists and chaplains) and provide guidelines in detail for how to collaborate, especially when those roles perceived differences in power. Further research should consider expanding our focus to other disciplines, such as psychologists, to gain a more comprehensive understanding of SDM within the entire PPC team and the collaborative patterns among team members.

### Strengths and limitations

To our knowledge, this is the first study to develop a SDM model in PPC.

Differences in culture, system and law between Western countries and non-Western countries may lead to certain differences in the details of SDM practice. Therefore, the transferability of the findings to other settings is uncertain.

## Conclusions

This study develops a model of SDM in PPC, which provides a structural approach to practice from three aspects: participants, principle and process. From a research perspective, the model contributes to the existing body of knowledge by providing a conceptual model for SDM in the context of PPC. From a clinical practice perspective, the model provides practical guidance for healthcare providers in PPC, promoting families’ autonomy and satisfaction while ensuring care plans align with their values.

### Electronic supplementary material

Below is the link to the electronic supplementary material.

**Supplementary Material 1**: Interview guide.

## Data Availability

The datasets used and/or analysed during the current study are available from the corresponding author on reasonable request.

## List of abbreviations

SDM: Shared decision-making
PPC: Pediatric palliative care
