# Correction: A shared decision-making model in pediatric palliative care: a qualitative study of healthcare providers

**DOI:** 10.1186/s12904-024-01361-2

**Published:** 2024-01-23

**Authors:** Siyu Cai, Lei Cheng, Ruixin Wang, Xuan Zhou, Xiaoxia Peng

**Affiliations:** 1 Center for Clinical Epidemiology and Evidence-Based Medicine, Beijing Children’s Hospital, Capital Medical University, National Center for Children’s Health, 56 South Lishi Road, Beijing, 100045 China; 2 School of Nursing, Fudan University, Shanghai, 200032 China; 3 Beijing Key Laboratory of Pediatric Hematology Oncology; National Key Discipline of Pediatrics (Capital Medical University); Key Laboratory of Major Diseases in Children, Ministry of Education; Hematology Center, Beijing Children’s Hospital, Capital Medical University, National Center for Children’s Health, 56 South Lishi Road, Beijing, 100045 China

**Correction: BMC Palliat Care 22, 190 (2023)**

**https://doi.org/10.1186/s12904-023-01307-0**

Following publication of the original article [1], the author reported that the equal contribution symbol has been assigned to the 3rd and 4th author when it should be for the 4th and the last author.

This has been corrected above and the original article has been updated.
