# Supplementary material for: A shared decision-making model in pediatric palliative care: a qualitative study of healthcare providers
Source: BMC Palliat Care. 2023 Nov 28;22:190. doi: 10.1186/s12904-023-01307-0 (PMC10683132; doi:10.1186/s12904-023-01307-0)
Supplement: Supplementary file 1 — Supplementary Material 1: Interview guide. [file 12904_2023_1307_MOESM1_ESM.docx]

INTERVIEW GUIDE

1. Please share your experience of SDM. Please provide examples of medical decisions and other decisions separately.

1. Which decisions are suitable for SDM?
2. What key points or principles need to be paid attention to in the process of SDM?

2. What are the steps involved in SDM in pediatric palliative care?

3. What difficulties have you encountered in the process of SDM? How did you address these difficulties?

4. Who may be involved in the process of SDM?

(1) What are their roles and responsibilities?

(2) How do the participants collaborate and interact? Are there specific communication and collaboration mechanisms in place?

5. Is there any other information about the SDM you would want to share with us?
